# Biosynthesis of selenium nanoparticles from *Dahlia pinnata* tuberous roots with antibacterial, antidiabetic, and erythrocyte membrane protective activities

**DOI:** 10.1038/s41598-025-12457-x

**Published:** 2025-07-26

**Authors:** Alsayed E. Mekky, Abdullah M. Abdo, Muhammed I. Haggag, Mohammed H. Elhaw, Mostafa M. Kadry, Sameh M. Ghanem, Mokhtar M. Salama, Amal M. Soliman, Nashaat N. Mahmoud

**Affiliations:** 1 Botany and Microbiology Department, Faculty of Science, Al-Azhar University, P.O. Box 11884, Nasr City, Cairo, Egypt; 2 Food Science and Technology Department, Faculty of Agriculture, Al-Azhar University, P.O. Box 13759, Nasr City, Cairo, Egypt; 3 Department of Medical Microbiology and Immunology, Faculty of Medicine, Ain-Shams University, P.O. Box 1181, Cairo, Egypt

**Keywords:** Antidiabetic potential, Antimicrobial activity, *Dahlia pinnata* L., *Escherichia coli*, Green synthesis, Selenium nanoparticles (SeNPs), Biological techniques, Biotechnology, Medical research

## Abstract

Selenium nanoparticles (SeNPs) synthesized using *Dahlia pinnata L* tuber extract exhibit significant antimicrobial, antidiabetic, and anti-inflammatory properties. This study describes the green synthesis of SeNPs using *D.* pinnata L. tuber extract, a sustainable approach that leverages plant-based compounds. Green synthesis was confirmed via UV-Vis spectroscopy (280 nm peak), XRD trigonal crystal structure with planes (100), (101), (110), (111), and (201), and TEM (spherical particles, 17.37 nm average size). FTIR revealed functional groups (C–H, C = O, Se–Se), while HPLC identified gallic acid (1070.58 µg/g) and chlorogenic acid (903.87 µg/g). In antimicrobial studies, SeNPs demonstrated strong efficacy against 70 *Escherichia coli* isolates from laboratory samples of diabetic patients. For 15 multidrug-resistant (MDR, 21.4%) isolates, SeNPs had MICs of 25–50 µg/ml (mean: 35 ± 12 µg/ml) and MBCs of 50–100 µg/ml (mean: 76.6 ± 26 µg/ml). Non-MDR isolates (78.6%) were more sensitive, with MICs of 10–25 µg/ml (mean: 15 ± 4.5 µg/ml) and MBCs of 25–50 µg/ml (mean: 35 ± 12 µg/ml), outperforming selenium precursors and vancomycin. SeNPs also showed antidiabetic potential through α-amylase inhibition (IC_50_ = 50.32 µg/ml) and α-glucosidase inhibition (IC_50_ = 31.55 µg/ml), though less effective than acarbose (IC_50_ = 5.85 µg/ml and 3.93 µg/ml, respectively). In anti-inflammatory assays, SeNPs achieved dose-dependent hemolysis inhibition, with 96.0% inhibition at 1000 µg/ml and an IC_50_ of 11.53 µg/ml, compared to indomethacin’s IC_50_ of 4.51 µg/ml. These findings demonstrate the promising in vitro bioactivities of SeNPs synthesized from *D. pinnata* L. tuber extract and support their potential for further preclinical investigation.

**Supplementary Information:**

The online version contains supplementary material available at 10.1038/s41598-025-12457-x.

## Introduction

Nanotechnology has emerged as a pivotal area of scientific innovation, offering cutting-edge solutions across various fields, including medicine, agriculture, and environmental sciences^1–3^. Within this domain, selenium nanoparticles (SeNPs) have garnered substantial attention due to their remarkable physicochemical properties and extensive biological applications^4,5^. Selenium (Se), a vital trace element required for numerous physiological functions, plays a crucial role in maintaining cellular health through its involvement in antioxidant defense, thyroid metabolism, and immune modulation^6–8^. Despite its essentiality, selenium can be toxic at higher concentrations, necessitating the development of safer and more bioavailable forms^9^. SeNPs have been shown to possess reduced toxicity compared to their bulk counterparts, making them an attractive alternative for biomedical applications^10^. The growing interest in SeNPs has catalyzed the exploration of eco-friendly and sustainable synthesis methods, particularly through plant-mediated green synthesis approaches^11,12^.

Plant-mediated synthesis of nanoparticles is a promising strategy that leverages the natural reducing and stabilizing agents present in plant extracts^1,13–16^. This method not only eliminates the need for hazardous chemicals but also incorporates bioactive compounds from plants into nanoparticles, thereby enhancing their biological activity^17^. *D. pinnata L.*, a widely cultivated ornamental plant, is recognized for its medicinal properties and the phytochemical richness of its tuberous roots^18,19^. These roots are known to contain an abundance of bioactive compounds, including phenolic acids, flavonoids, tannins, and saponins, which can serve as natural capping and stabilizing agents during nanoparticle synthesis^20^. Harnessing the phytochemical potential of plants in the biosynthesis of SeNPs represents a sustainable approach that combines green chemistry with enhanced therapeutic outcomes^21^.

One of the critical applications of biosynthesized SeNPs lies in their antimicrobial properties^10,11^. The alarming rise in multidrug-resistant (MDR) pathogens poses a significant threat to global health, necessitating the search for alternative antimicrobial agents^22^. SeNPs have demonstrated efficacy against a wide range of pathogens, including bacteria, fungi, and viruses, through mechanisms such as the generation of reactive oxygen species (ROS), disruption of microbial membranes, and interference with intracellular signaling pathways^11,22,23^. By incorporating the bioactive compounds from *D. pinnata L* during the synthesis process, the antimicrobial potential of SeNPs can be further amplified, providing a robust solution to combat MDR infections^20^.

In addition to antimicrobial activity, the therapeutic potential of SeNPs extends to the management of diabetes mellitus, a chronic metabolic disorder characterized by hyperglycemia and associated complications^4,24^. Oxidative stress and inflammation play pivotal roles in the pathogenesis of diabetes and its complications, including cardiovascular disease, neuropathy, and nephropathy^25^. SeNPs have shown promise in mitigating oxidative damage and improving glucose metabolism by enhancing the activity of endogenous antioxidant enzymes and modulating inflammatory pathways^26,27^. The functionalization of SeNPs with bioactive plant-derived compounds may further enhance their efficacy in regulating blood sugar levels and improving insulin sensitivity, offering a potential adjunctive therapy for diabetes management^28^.

Another compelling aspect of SeNPs is their ability to protect erythrocyte membrane integrity^29^. Erythrocytes, or red blood cells, are essential for oxygen transport but are highly susceptible to oxidative damage due to their high polyunsaturated lipid content and continuous exposure to reactive oxygen species (ROS)^30^. Oxidative stress can compromise erythrocyte membrane integrity, leading to hemolysis and impaired oxygen delivery to tissues^31^. SeNPs, owing to their potent antioxidant properties, can prevent such damage by neutralizing free radicals and reducing lipid peroxidation. When synthesized using *D. pinnata L* extracts, SeNPs may provide additional protective effects through the synergistic action of phytochemicals, ensuring enhanced stability and resilience of erythrocyte membranes^10^.

This study is centered on evaluating the bioactive potential of selenium nanoparticles (SeNPs) synthesized from extracts of the tuberous roots of *D. pinnata L*. The primary objectives include assessing their antimicrobial activity against clinically relevant *E. coli* pathogens, investigating their role in diabetes management through α-glucosidase and α-amylase inhibition assays, and evaluating their protective effects on erythrocyte membrane integrity against hemolytic damage. By focusing on these key aspects, this research aims to provide valuable insights into green nanotechnology and the applications of SeNPs in medicine and their potential therapeutic benefits.

## Materials and methods

### Materials

Plant Materials: Fresh *D. pinnata L.* roots were collected from Orman Botanical Garden, Giza. Plant collection was conducted in compliance with local and international regulations. Permission for collecting *Dahlia pinnata* L. was obtained from the Botany and Microbiology department, Faculty of Science, Al-Azhar University, Cairo, Egypt, and the plant was identified and used according to institutional guidelines. The formal identification of *Dahlia pinnata* L. has been conducted by Dr. Mohammed Hagag, Assistant Professor of Botany, Faculty of Science, Botany and Microbiology Department, Cairo, Egypt. A voucher specimen has been accepted for deposit at the Herbarium of the Botany and Microbiology Department, under the Deposition Code: AZU/SCI/BOT/HERB/2025 − 101. The specimen is publicly available for future reference following the herbarium’s policies. The plant material collected at site coordinate: 30°01’43.8"N 31°12’45.9"E, Egypt, was cleaned, air-dried, and powdered using an IKA Multidrive Basic Grinder (IKA, Germany).

**Fig. 1:**
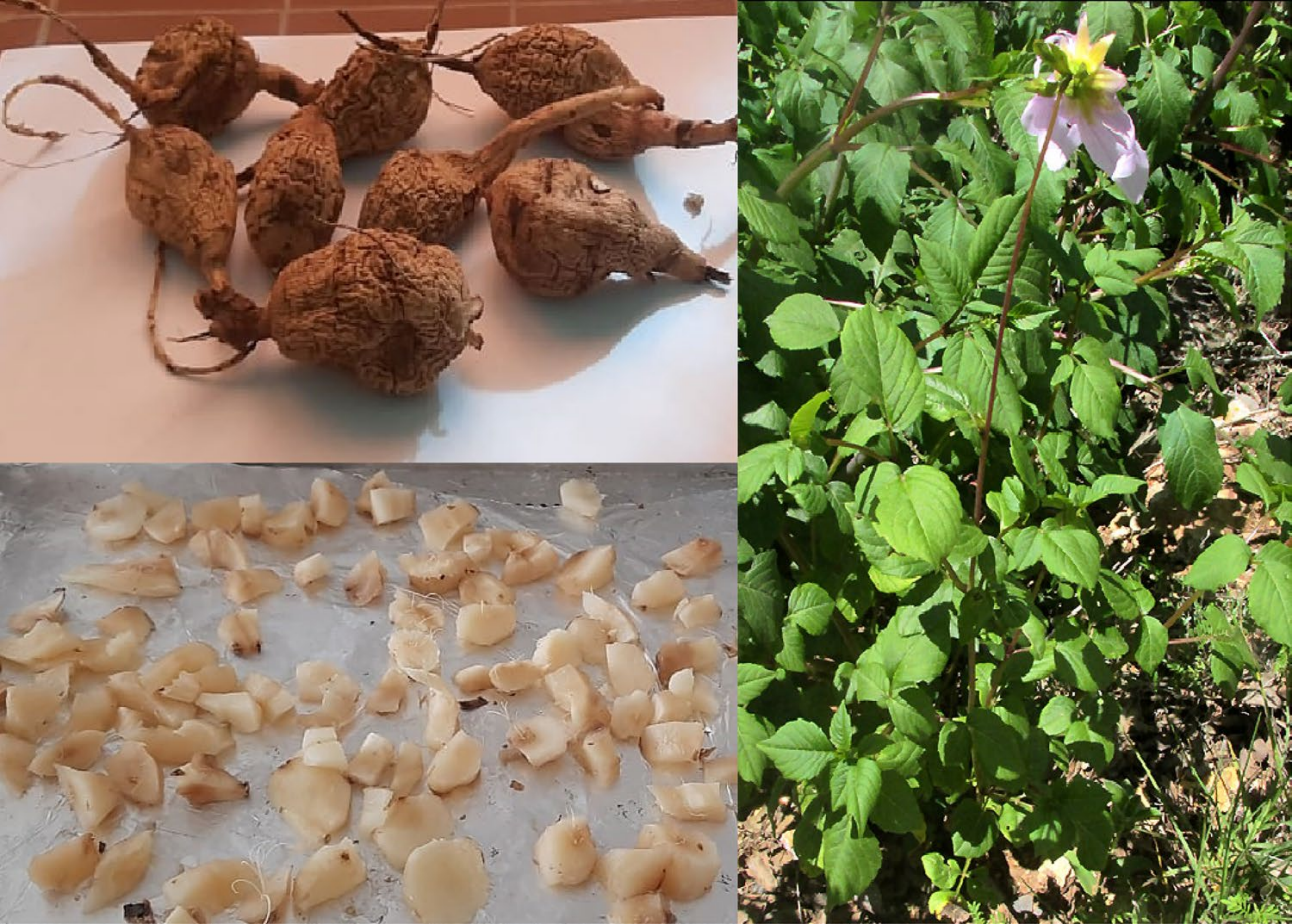
*Dahlia pinnata L.* tuberous roots.

Aqueous extracts were prepared by heating 10 g of root powder with 100 ml of deionized water at 70 °C, followed by filtration through Whatman No. 1 filter paper (Cytiva, USA), Fig. 1.

Isolates: Seventy *E. coli* isolates were obtained from laboratory samples of diabetic patients at Ain Shams University Hospital. Samples (urine, wound swabs, blood, and sputum) were cultured on MacConkey agar and Blood agar (HI Media, India) and incubated at 37 °C for 24 h. Suspected isolates were confirmed through biochemical tests and the API 20E Identification System (bioMérieux, France). Isolates were classified as multidrug-resistant (MDR) or non-MDR.

Antibiotic Discs: Vancomycin (30 µg), clindamycin (2 µg), penicillin (10 IU), amoxicillin-clavulanate (20/10 µg), and tetracycline (30 µg) discs were procured from Oxoid Ltd. (UK) and tested on Mueller-Hinton Agar (HI Media, India) following CLSI guidelines.

Reagents and Chemicals: Sodium selenite (≥ 99%, Sigma-Aldrich, USA), ethanol (analytical grade), and sodium hydroxide (≥ 98%, Merck, Germany) were used. Deionized water (18.2 MΩ·cm) was prepared using an Elga Purelab System (Veolia, USA). All chemicals and consumables were of analytical grade.

### Preparation of *Dahlia pinnata L.* extract

The collected tuberous roots were thoroughly washed under running tap water to remove soil and debris. Subsequently, they were rinsed three times with deionized water to ensure complete removal of surface contaminants. The clean roots were air-dried at room temperature (approximately 25 °C) for 72 h in a shaded area to prevent photodegradation of bioactive compounds. After drying, the roots were cut into smaller pieces and ground into a fine powder using a mechanical grinder (IKA Multidrive Basic, Germany).

For extract preparation, 10 g of the powdered material was weighed using a high-precision analytical balance (Sartorius Entris II, Germany) and added to 100 ml of deionized water in a sterile 250 ml Erlenmeyer flask. The mixture was heated at 70 °C for 30 min on a magnetic stirrer-hot plate (IKA C-MAG HS7, Germany) with constant stirring at 300 rpm. The resulting solution was allowed to cool to room temperature and then filtered through Whatman No. 1 filter paper (pore size 11 μm). The filtrate, containing bioactive compounds, was collected in sterile amber glass bottles and stored at 4 °C for further use.

### Biosynthesis of selenium nanoparticles

The biosynthesis of selenium nanoparticles was carried out by mixing the prepared *D. pinnata L* extract with an aqueous sodium selenite solution. 1 mM sodium selenite solution was freshly prepared by dissolving 0.172 g of sodium selenite in 1 L of deionized water. In a typical reaction, 50 ml of *D. pinnata L* extract was mixed with 50 ml of 1 mM sodium selenite solution in a 250 ml Erlenmeyer flask. The pH of the reaction mixture was adjusted to 8 using a 0.1 M sodium hydroxide solution, prepared by dissolving 4 g of sodium hydroxide pellets in 1 L of deionized water. The reaction mixture was incubated at room temperature (25 ± 2 °C) on a magnetic stirrer set to 200 rpm for 24 h. A visible color change from pale yellow to reddish orange was observed, indicating the reduction of selenium ions and the formation of selenium nanoparticles.

### Purification of selenium nanoparticles

The synthesized selenium nanoparticles were purified to remove unreacted precursors and other organic residues. The reaction mixture was transferred to 50 ml centrifuge tubes and centrifuged at 10,000 rpm for 20 min at 4 °C using a refrigerated centrifuge (Eppendorf 5810R, Germany). The resulting reddish-orange pellet was washed three times with deionized water and once with 70% ethanol to ensure the removal of impurities. After washing, the purified nanoparticles were dried in a vacuum oven (Memmert VO400, Germany) at 50 °C for 24 h. The dried nanoparticles were carefully collected, weighed, and stored in a sterile glass container at room temperature for subsequent characterization.

### Characterization of selenium nanoparticles

#### Color change observation method for characterization of selenium nanoparticles (SeNPs)

The synthesis of selenium nanoparticles (SeNPs) from *D. pinnata L* tuberous root extract was characterized by monitoring the visual color change that occurs during the biosynthesis process, a common feature in nanoparticle formation. The color change is an indicative visual marker of nanoparticle formation and can be attributed to surface plasmon resonance (SPR) effects, which are characteristic of metal nanoparticles, including selenium.

#### UV-visible spectroscopy

The optical properties of the biosynthesized selenium nanoparticles were analyzed using a UV-visible spectrophotometer (Shimadzu UV-1800, Japan). The reaction mixture was scanned in the wavelength range of 200–800 nm at a resolution of 1 nm to identify the characteristic surface plasmon resonance (SPR) peak of selenium nanoparticles. The presence of a peak in the range of 260–320 nm was indicative of selenium nanoparticle formation. Baseline correction was performed using deionized water, and the spectra was recorded in triplicate to ensure reproducibility.

#### Fourier transform infrared (FTIR) spectroscopy

FTIR analysis was performed using a PerkinElmer Spectrum Two spectrometer (USA) to identify the functional groups involved in the reduction of selenium ions and the capping/stabilization of the nanoparticles by biomolecules in the *D. pinnata L* extract. The dried selenium nanoparticles were mixed with potassium bromide (KBr) in a 1:100 ratio, pressed into a pellet, and scanned in the mid-infrared region (4000–400 cm^–1^) at a resolution of 4 cm^–1^. Peaks corresponding to hydroxyl (-OH), carbonyl (C = O), and amine (-NH) groups were analyzed to confirm their role in nanoparticle stabilization.

#### Transmission electron microscopy (TEM)

The detailed internal structure and exact size of the selenium nanoparticles were further analyzed using a TEM instrument (JEOL JEM-2100, Japan) operating at 200 kV. A drop of SeNPs suspension was placed on a carbon-coated copper grid and air-dried before imaging. High-resolution TEM (HRTEM) was used to confirm the crystalline nature of the nanoparticles, and selected area electron diffraction (SAED) patterns were recorded to examine their lattice structure.

#### X-ray diffraction (XRD)

The crystalline structure and phase purity of the selenium nanoparticles were determined using a Bruker D8 Advance X-ray diffractometer (Germany) equipped with Cu Kα radiation (λ = 1.5406 Å). The dried SeNPs were placed on a sample holder, and XRD patterns were recorded in the 2θ range of 10°–80° at a scanning rate of 1°/min. The diffraction peaks were matched with standard reference data from the Joint Committee on Powder Diffraction Standards (JCPDS) database to identify the crystal structure of selenium. The average crystallite size was calculated using the Debye-Scherrer equation.

#### Preparation of plant extract for HPLC analysis

The tuberous roots of *D. pinnata L* were collected, washed thoroughly with distilled water to remove any dirt or debris, and air-dried under shade at room temperature for 10 days. The dried roots were ground into a fine powder using an electric grinder (Panasonic MX-AC400, Japan). To prepare the extract, 2 g of the powdered root material was mixed with 20 ml of methanol (HPLC grade, Sigma-Aldrich, USA) in a 50 ml conical flask. The extraction process was enhanced using an ultrasonic bath (Branson Ultrasonics, USA) operated at 40 kHz and a temperature of 40 °C for 30 min. The resulting mixture was filtered through Whatman No. 1 filter paper, followed by further filtration through a 0.45 μm syringe filter (Millipore, USA). The filtered extract was collected in amber glass vials and stored at 4 °C to prevent degradation until further analysis.

#### High-performance liquid chromatography (HPLC) analysis

Quantitative analysis of phenolic and flavonoid compounds in the *D. pinnata L.* root extract was performed using a High-Performance Liquid Chromatography (HPLC) system (Agilent 1260 Infinity II) equipped with a diode-array detector (DAD). The chromatographic separation was carried out on an Agilent ZORBAX Eclipse Plus C18 column (4.6 × 150 mm, 5 μm particle size), which was maintained at a constant temperature of 30 °C. The mobile phase consisted of two solvents: solvent A (0.1% formic acid in water) and solvent B (acetonitrile, HPLC grade, Sigma-Aldrich, USA). A gradient elution program was employed, starting with 10% solvent B and increasing to 90% solvent B over 30 min, followed by a return to initial conditions. The total running time was 35 min with a flow rate of 1 ml/min. Detection wavelengths were set to 280 nm for phenolics and 330 nm for flavonoids, based on their maximum absorption.

#### Preparation of standards

Reference standards for the following compounds were procured from Sigma-Aldrich (USA) with ≥ 98% purity: gallic acid, chlorogenic acid, catechin, methyl gallate, caffeic acid, syringic acid, pyro catechol, rutin, ellagic acid, coumaric acid, vanillin, ferulic acid, naringenin, rosmarinic acid, daidzein, quercetin, cinnamic acid, kaempferol, and hesperetin. Standard solutions were prepared in methanol at concentrations ranging from 10 to 100 µg/ml. Each standard was individually injected into the HPLC under the same chromatographic conditions as the sample to determine retention times and generate calibration curves. Calibration curves were constructed by plotting peak areas against the known concentrations of each standard, and linear regression analysis was performed to validate their linearity (R² > 0.99).

#### Analysis of plant extract

The filtered *D. pinnata L* extract was analyzed using the same HPLC conditions as the standards. Peaks in the chromatogram were identified by comparing their retention times with those of the standards. Quantification of individual compounds was achieved by calculating their concentrations (µg/ml) using the calibration curves. The results were further normalized to concentrations per gram of the powdered extract (µg/g) by accounting for the sample weight and volume of solvent used.

### Biological activity

#### Antimicrobial assay

This pilot study was conducted from July 2024 to January 2025 on seventy *E. coli* isolates collected from diabetic patients presenting with urinary tract infections, wound infections, bloodstream infections, and respiratory tract infections obtained from inpatient and outpatient clinical samples submitted to the central microbiology laboratory at Ain Shams University Hospital. These samples were collected using sterile techniques to avoid contamination and transported immediately to the microbiology laboratory.

Upon receipt, the samples were cultured on MacConkey agar and Blood agar and incubated aerobically at 37 °C for 24 h. Colonies suspected of being *E. coli* were identified based on colony morphology (pink, lactose-fermenting colonies on MacConkey agar), Gram staining (Gram-negative bacilli), and a series of biochemical tests, including oxidase, catalase, methyl red, Voges-Proskauer, citrate utilization, and triple sugar iron agar tests. Final confirmation was performed using the API 20E identification system.

Isolates were categorized based on their resistance patterns according to international consensus criteria for multidrug resistance (MDR), into MDR isolates that are resistant to at least one agent in three or more antibiotic classes. This includes β-lactams (e.g., penicillin, cephalosporin), aminoglycosides, and tetracyclines. MDR strains pose significant treatment challenges due to their broad resistance. Non-MDR isolates are susceptible to agents in at least two or more antibiotic classes and are resistant to fewer than three classes.

The inclusion criteria were bacterial isolates that were confirmed to be *E coli* recovered from different types of infections of diabetic patients, and the exclusion criteria were bacterial isolates other than *E coli* or from non-diabetic patients.

#### Antimicrobial susceptibility testing

The antimicrobial susceptibility of all isolates was tested using the Kirby-Bauer disk diffusion method on Mueller-Hinton agar, following CLSI guidelines. The following antibiotics were selected based on their clinical relevance: Vancomycin (VA, 30 µg), Clindamycin (CLI, 2 µg), Penicillin (PEN, 10 IU), Amoxicillin-clavulanate (AMC, 20/10 µg), Tetracycline (TET, 30 µg) .

A standardized bacterial suspension equivalent to a 0.5 McFarland turbidity standard (1 × 10⁸ CFU/ml) was prepared from fresh overnight cultures. The suspension was uniformly spread over Mueller-Hinton agar plates using a sterile swab. Antibiotic disks were placed on the agar surface using sterile forceps, and plates were incubated at 37 °C for 18–24 h. After incubation, the diameter of the inhibition zones around each disk was measured in millimeters. Results were classified as sensitive (S), intermediate (I), or resistant (R) based on CLSI breakpoints.

#### Preparation of bacterial cultures

The bacterial strain *E. coli* was revived from glycerol stocks and cultured in nutrient broth (Oxoid, UK) at 37 °C for 18–24 h. Fresh overnight cultures were adjusted to a turbidity equivalent to 0.5 McFarland standard (~ 1.5 × 10⁸ CFU/ml) and used for the antimicrobial assays.

#### Disk diffusion method

Antimicrobial activity was assessed using the disk diffusion method as per the Clinical and Laboratory Standards Institute (CLSI, 2024) guidelines. Sterile Mueller-Hinton agar (MHA) plates were inoculated with standardized bacterial suspensions using sterile cotton swabs. Sterile 6-mm filter paper disks were impregnated with 20 µl of *D. pinnata L* root extract (10 mg/ml) or biosynthesized SeNPs (5 mg/ml) and placed on the agar surface. Disks with sterile distilled water and ciprofloxacin (5 µg, Sigma-Aldrich) served as negative and positive controls, respectively. Plates were incubated at 37 °C for 24 h, and the diameter of the inhibition zones (in mm) was measured.

#### Minimum inhibitory concentration (MIC) and minimum bactericidal concentration (MBC)

The MIC of selenium precursors (sodium selenite) and reference antibiotics were determined using the broth microdilution method in 96-well plates. Serial twofold dilutions of the test samples were prepared in Mueller-Hinton broth, each well was inoculated with 100 µl of bacterial suspension (final concentration: 1 × 10⁶ CFU/ml), and incubated at 37 °C for 24 h. The MIC was defined as the lowest concentration of the test sample that inhibited visible growth after 24 h of incubation. MBC was determined by plating aliquots from wells with no visible growth onto Mueller-Hinton Agar MHA plates. The lowest concentration resulting in no bacterial growth was recorded as the MBC; MIC: The MIC was defined as the lowest concentration that inhibited visible bacterial growth. While determining the MBC, aliquots from wells showing no growth were sub-cultured onto Mueller-Hinton agar plates. The MBC was the lowest concentration that resulted in ≥ 99.9% bacterial killing.

### Diabetes management

#### α-glucosidase inhibitory assay

The inhibitory activity of *D. pinnata L* tuberous root extracts on α-glucosidase enzyme was assessed using a method adapted from Pistia Brueggeman and Hollingsworth (2001), with slight modifications. The α-glucosidase enzyme plays a key role in the breakdown of carbohydrates into glucose and is a target for inhibiting postprandial hyperglycemia in diabetic patients. In this assay, plant extracts were tested for their ability to inhibit α-glucosidase activity, which could potentially contribute to glucose regulation.

The assay was carried out in a 96-well microplate format. Plant extracts were prepared in 0.1 M phosphate buffer (pH 6.8) at concentrations ranging from 1.97 to 1000 µg/ml through serial dilution. In each well, 50 µl of the prepared plant extract was mixed with 10 µl of α-glucosidase enzyme solution (1 U/ml, obtained from Sigma-Aldrich, St. Louis, MO, USA). The reaction mixture was then adjusted with 125 µl of 0.1 M phosphate buffer to achieve a total volume of 185 µl per well. The enzyme extract mixture was incubated at 37 °C for 20 min, allowing the enzyme to interact with the plant extract.

After the 20-minute incubation, the reaction was initiated by adding 20 µl of 1 M p-nitrophenyl-α-D-glucopyranoside (pNPG), a specific substrate for α-glucosidase. This substrate is hydrolyzed by the enzyme to release p-nitrophenol, which can be quantitatively measured. The reaction mixture was then incubated for an additional 30 min at 37 °C, providing sufficient time for the enzymatic hydrolysis of the substrate.

To terminate the reaction, 50 µl of 0.1 N sodium carbonate (Na₂CO₃) was added to each well. This addition stops the enzymatic reaction by increasing the pH and deactivating the enzyme. The absorbance of the resulting solution, which is proportional to the amount of p-nitrophenol produced, was measured at 405 nm using a Biosystem 310 Plus spectrophotometer (Biosystem, Barcelona, Spain). The absorbance readings were used to calculate the percentage inhibition of α-glucosidase activity.

The percentage inhibition of the enzyme was determined using the formula^32^:

$$Glucosidase~inhibition~\% ~=100 - \left( {\frac{{O{D_{BLANK}} - O{D_{SAMPLE}}}}{{O{D_{Blank}}}}~x~100} \right)$$

Where OD _BLANK_ is the absorbance of Enzyme without samples, and OD _SAMPLE_ is the absorbance of enzyme with sample (in the presence of the plant extract).

The assay provides an estimate of the inhibitory effect of the plant extract on the enzyme’s ability to hydrolyze the substrate. One unit of the enzyme can be defined as the amount of enzyme (α-glucosidase) required for the formation of one µmol of product (p-Nitrophenol) from the substrate (*p*-nitrophenyl-*α*-D-glucopyranoside) per minute. To further quantify the potency of the plant extract, the IC_50_ value was determined. The IC_50_​ is the concentration of extract required to inhibit 50% of the enzyme’s activity. The IC_50_ was calculated using regression equation obtained through plotting concentration in the range 1.95–1000 µg/ml and % inhibition for different extracts and fractions.

#### In vitro α-amylase inhibitory assay

The inhibitory activity of *D. pinnata L* tuberous root extract on α-amylase was evaluated using the 3,5-dinitrosalicylic acid (DNSA) method, a standard technique for measuring the hydrolysis of starch by α-amylase. The assay was designed to assess the ability of the plant extract to inhibit the enzymatic breakdown of starch, which is important for controlling blood glucose levels, especially in the context of diabetes management.

To begin, the plant extract was prepared by dissolving a small amount of the extract in 10% dimethyl sulfoxide (DMSO), ensuring it was fully dissolved. This stock solution was then further diluted with a buffer solution containing 0.02 M sodium hydrogen phosphate (Na₂HPO₄), 0.02 M sodium dihydrogen phosphate (NaH₂PO₄), and 0.006 M sodium chloride (NaCl) to achieve a series of concentrations ranging from 1.9 to 1000 µg/ml. The buffer solution was adjusted to pH 6.9, which is optimal for the α-amylase enzyme activity.

In the assay, 200 µl of α-amylase solution (2 units/ml, sourced from a commercial supplier) was mixed with 200 µl of the prepared plant extract at varying concentrations. This mixture was incubated for 10 min at 30 °C to allow the extract to interact with the enzyme and initiate any potential inhibitory effects on α-amylase activity. After the incubation period, 200 µl of a 1% starch solution (w/v) was added to each tube to serve as the substrate for the α-amylase enzyme. The starch solution was prepared in distilled water to ensure a consistent substrate concentration across all samples. The reaction mixture was incubated for an additional 3 min to allow the enzyme to catalyze the breakdown of starch into smaller sugar molecules.

To stop the enzymatic reaction, 200 µl of 3,5-dinitrosalicylic acid (DNSA) reagent was added to each reaction tube. The DNSA reagent was prepared by dissolving 12 g of sodium potassium tartrate tetrahydrate in 8.0 ml of 2 M sodium hydroxide (NaOH) and combining it with 20 ml of 96 mM 3,5-dinitrosalicylic acid solution. This reagent reacts with reducing sugars released during starch hydrolysis to form a red-colored complex, the intensity of which is proportional to the amount of sugar produced. The reaction mixture was then heated in a water bath at 85–90 °C for 10 min to promote the color development. After boiling, the mixture was allowed to cool to ambient temperature, and then 5 ml of distilled water was added to dilute the reaction mixture, ensuring the absorbance measurement would fall within the detectable range.

The absorbance of the resulting solution was measured at 540 nm using a UV-Visible Biosystem 310 spectrophotometer. The control reaction, representing 100% enzyme activity, was prepared by replacing the plant extract with an equal volume of buffer. Additionally, a blank reaction was prepared using the plant extract in the absence of the enzyme to account for any absorbance due to the extract itself. The α-amylase inhibitory activity was then calculated by comparing the absorbance of the sample reactions to that of the control. The percent inhibition of α-amylase was determined using the following equation^33^:

$$~\alpha - amylase~inhibition~\% ~=\frac{{\left( {Ab{s_{control}} - Ab{s_{sample}}} \right)}}{{Ab{s_{control}}}}~ \times 100$$

Where Abs _control_ is the absorbance of the control reaction, and Abs _sample_ ​ is the absorbance of the reaction containing the plant extract. This equation reflects the ability of the plant extract to inhibit the α-amylase enzyme, with higher inhibition values indicating greater inhibitory potential.

To further quantify the inhibitory potency of the plant extract, the IC_50_​ value was calculated. The IC_50_​ represents the concentration of the extract required to inhibit 50% of the enzyme activity and was obtained by plotting the percentage of α-amylase inhibition against the extract concentration. The resulting data were used to determine the concentration at which the extract exhibits half-maximal inhibition of the enzyme, providing a quantitative measure of its inhibitory strength.

### Evaluation of hemolytic activity

#### Preparation for erythrocyte suspension

Fresh whole blood was collected from healthy volunteers (3 ml) into heparinized tubes to prevent clotting. The blood was then subjected to centrifugation at 3000 rpm for 10 min to separate the blood components. Following centrifugation, the supernatant (plasma) was carefully removed, and the red blood cell (RBC) pellets were collected. An equal volume of normal saline was added to the RBC pellets to dissolve them. The volume of the dissolved RBCs (supernatant) was measured, and the final erythrocyte suspension was reconstituted to a 40% v/v suspension with an isotonic buffer solution.

The isotonic buffer was prepared by dissolving 0.2 g of NaH_2_PO_4_, 1.15 g of Na_2_HPO_4_, and 9 g of NaCl in 1 L of distilled water, resulting in a 10 mM sodium phosphate buffer at pH 7.4. The buffer ensures that the erythrocyte suspension remains at an osmolarity compatible with that of human blood, preventing cell lysis due to osmotic imbalance. The reconstituted erythrocytes (suspended red blood cells in the buffer) were gently mixed and stored at room temperature for immediate use in the hemolysis assay.

#### Hypotonicity-induced hemolysis assay

The hemolysis assay was used to evaluate the protective effects of the biosynthesized selenium nanoparticles (SeNPs) against erythrocyte lysis induced by hypotonic stress. For this assay, the SeNPs were first dissolved in distilled water to prepare a hypotonic solution. Various concentrations of the SeNPs (100, 200, 400, 600, 800, and 1000 µg/ml) were prepared by diluting the stock solution with distilled water. Additionally, a graded series of concentrations (3.9–1000 µg/ml) of the positive control, indomethacin, was also prepared in distilled water.

Two sets of centrifuge tubes were prepared for each treatment: one set containing the hypotonic solution (distilled water) and the other containing the isotonic solution (the 10 mM sodium phosphate buffer at pH 7.4). Each of the solutions (hypotonic and isotonic) was mixed with the corresponding graded doses of SeNPs or indomethacin. For each treatment, duplicate tubes were prepared for consistency.

A fixed volume (0.1 ml) of the erythrocyte suspension (40% v/v) was added to each centrifuge tube, and the tubes were gently mixed to ensure uniform distribution of the erythrocytes. The mixtures were incubated at room temperature (37 °C) for 1 h to allow for the interaction between the SeNPs and the erythrocytes, followed by centrifugation at 1300 g for 3 min to separate the supernatant from the erythrocyte pellet.

The extent of hemolysis was quantified by measuring the absorbance of the hemoglobin released into the supernatant at 540 nm using a Spectronic (Milton Roy) spectrophotometer. The absorbance of the supernatant reflects the amount of hemoglobin released due to erythrocyte membrane disruption.

#### Calculation of hemolysis and Inhibition of hemolysis

The percentage of hemolysis was calculated by comparing the absorbance of the test samples with that of the control samples. The hemolysis induced by distilled water (hypotonic solution) was assumed to represent 100% hemolysis, as it causes complete lysis of the erythrocytes.

To determine the inhibitory activity of the SeNPs on hemolysis, the percentage inhibition of hemolysis was calculated as follows^34^:

$${\text{Inhibition~of~Hemolysis~\% }}\,{\text{~}}=1 - \left( {\frac{{{\text{O}}{{\text{D}}_2} - {\text{O}}{{\text{D}}_1}}}{{{\text{O}}{{\text{D}}_3} - {\text{O}}{{\text{D}}_1}}}} \right) \times 100$$

Where: OD₁ is the absorbance of the test sample in isotonic solution (without hypotonic stress), OD₂ is the absorbance of the test sample in hypotonic solution (inducing hemolysis), and OD₃ is the absorbance of the control sample in hypotonic solution (positive control with complete hemolysis).

This equation quantifies the ability of SeNPs to protect the erythrocytes from osmotic lysis. The higher the percentage inhibition, the greater the protective effect of the SeNPs.

#### Data analysis and statistical evaluation

All experiments were conducted in triplicate to ensure reproducibility. The data were expressed as mean ± standard deviation (SD). The relative abundance of each compound in the extract was calculated as a percentage of the total phenolic and flavonoid content. Statistical analyses were performed using GraphPad Prism 9.0 software, with significant differences between compounds determined using one-way analysis of variance (ANOVA) followed by Tukey’s post hoc test. A *p-value* < 0.05 was considered statistically significant. Details are provided in Supplementary Information (see Supplementary Statistical Analysis 1).

## Results and discussion

### Synthesis of selenium nanoparticles (SeNPs)

The synthesis of selenium nanoparticles (SeNPs) using *D. pinnata L* tuberous root methanol extract was marked by a distinct and gradual color change in the solution, signaling the reduction of selenite ions (SeO_3_^2–^) into elemental selenium. Initially colorless or pale yellow, the solution turned reddish-brown or dark brown due to the surface plasmon resonance (SPR) effect of selenium nanoparticles. Similar findings were reported by Behera et al. (2024)^35^ who used plant extracts to synthesize SeNPs and observed a characteristic reddish-brown coloration, attributed to SPR effects. Ikram et al. (2024)^36^ noted the role of flavonoid-rich extracts in influencing reaction kinetics and color intensity during SeNPs synthesis.

**Fig. 2:**
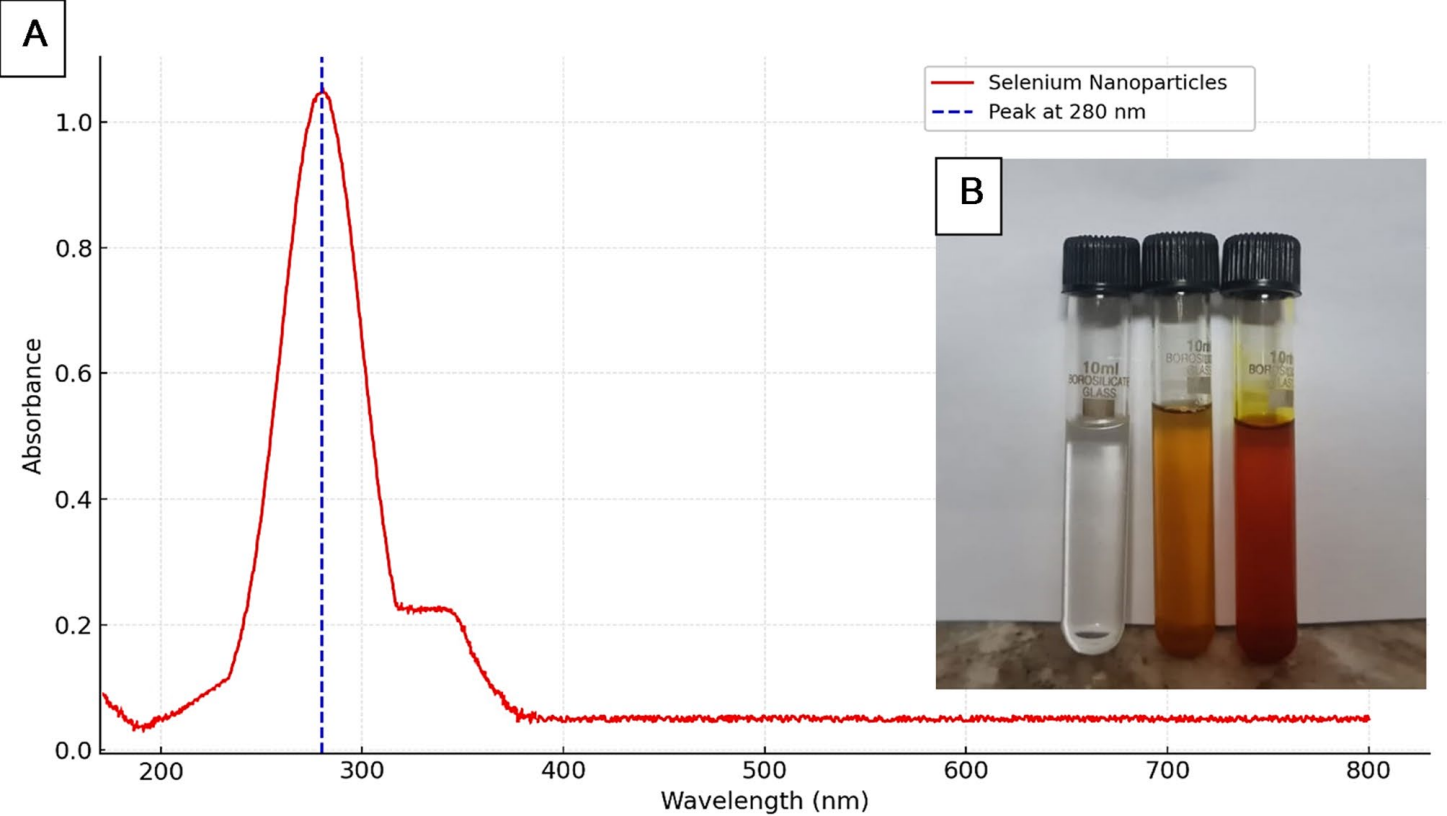
Color change observation and UV-visible spectroscopy of *D. pinnata L* tuberous roots photosynthesized selenium nanoparticles.

UV-Vis spectroscopy further confirmed the successful synthesis of SeNPs, with a characteristic SPR absorption peak at approximately 280 nm Fig. 2. This aligns with the findings of Satpathy et al. (2024)^37^ who reported SPR peaks at 290 nm using *Nyctanthes arbor-tristis* extract. Ikram et al. (2024)^36^ observed a peak at 275 nm, attributing variations in SPR to differences in nanoparticle size and stabilization by biomolecules in plant extracts. The presence of phenolics and flavonoids in the Dahlia tuber extract likely influenced these optical properties, as similarly noted in studies by Mamidi et al. (2024)^38^.

### Structural and morphological analysis of senps

**Fig. 3:**
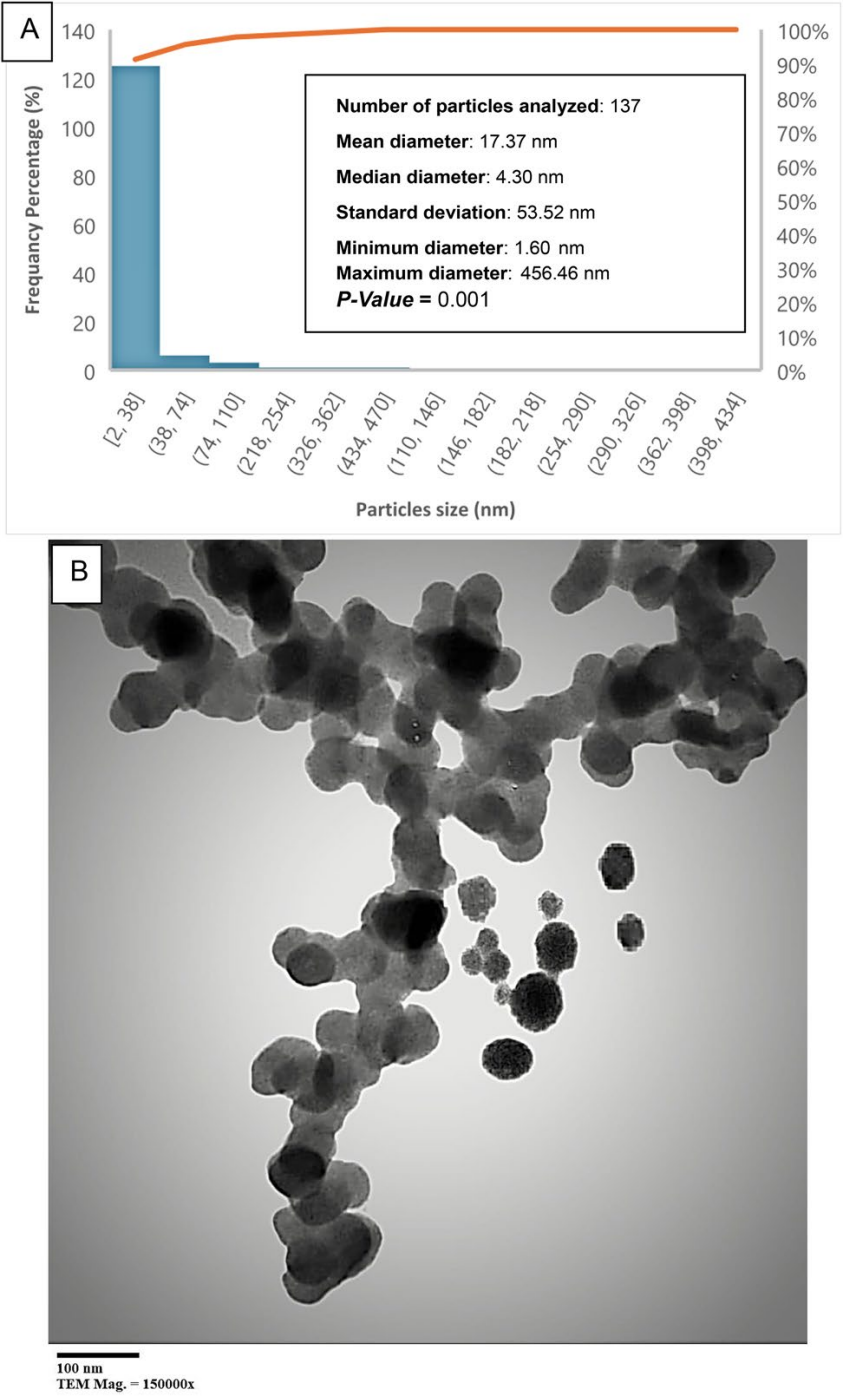
XRD Pattern of *D. pinnata L* tuberous roots phyto-synthesized (SeNPs).

X-ray diffraction (XRD) analysis revealed sharp peaks corresponding to the (100), (101), (110), (111), and (201) planes of selenium, confirming a trigonal crystal structure (JCPDS card no. 03-065-3883). A slight peak broadening indicated nanoscale particle dimensions Fig. 3. These results align with Satpathy et al. (2024)^37^ and Mamidi et al. (2024)^38^ who also identified similar XRD patterns, with minor variations linked to precursor concentrations and capping agents.

**Fig. 4:**
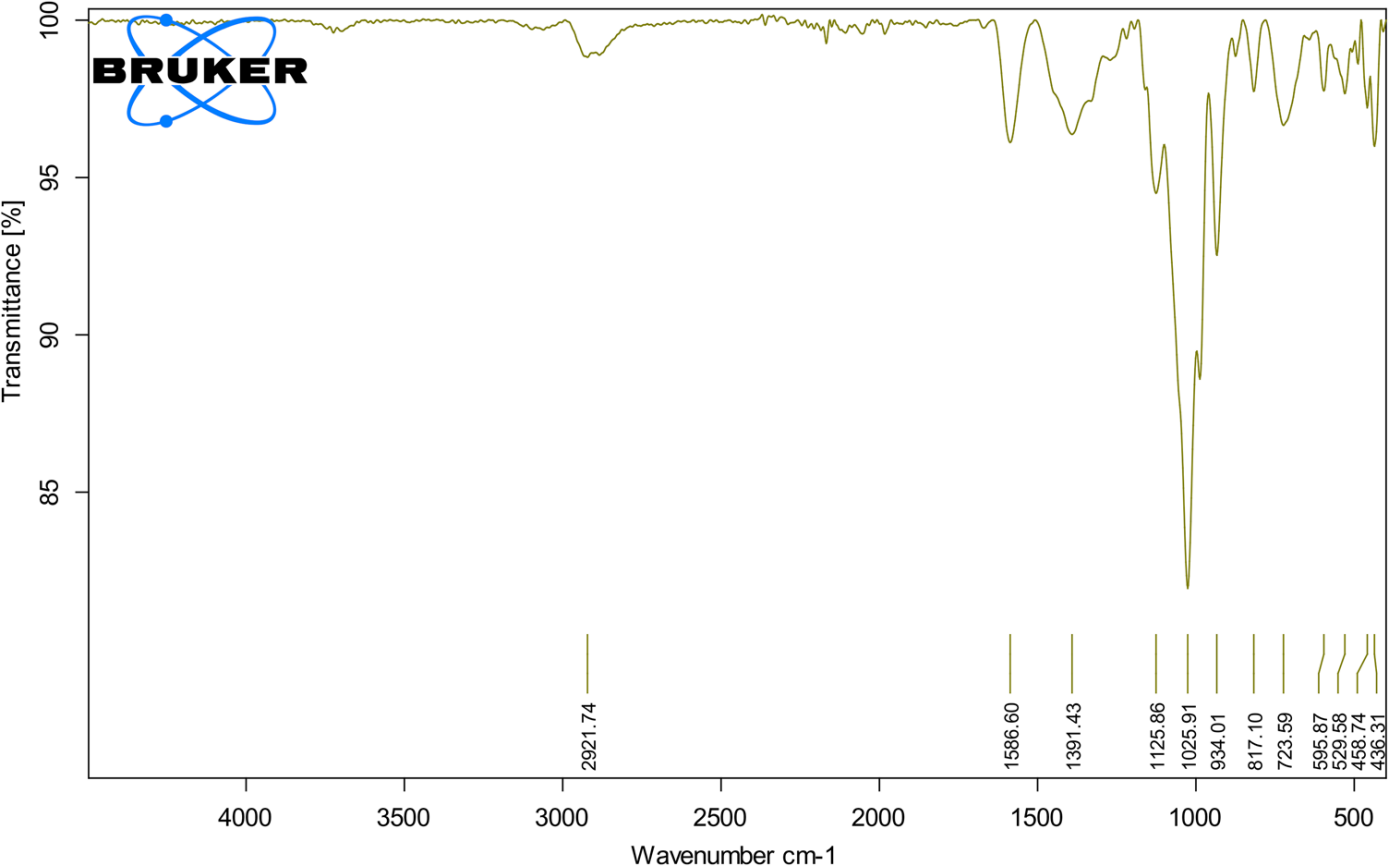
Particle size distribution and TEM image of *D. pinnata L* tuberous roots, phyto-synthesized (SeNPs).

Transmission Electron Microscopy (TEM) revealed predominantly spherical SeNPs with a mean diameter of 17.37 nm and a median diameter of 4.30 nm. The size distribution indicated most particles were below 20 nm, with a standard deviation of 53.71 nm Fig. 4. Aggregation, commonly observed in biogenic SeNPs, was attributed to biomolecules acting as reducing and stabilizing agents. Mamidi et al. (2024)^38^ and Ikram et al. (2024)^36^. Similarly reported spherical SeNPs with sizes between 10 and 50 nm, with narrower distributions, were achieved by optimizing synthesis conditions.

### FTIR analysis and role of biomolecules

**Fig. 5:**
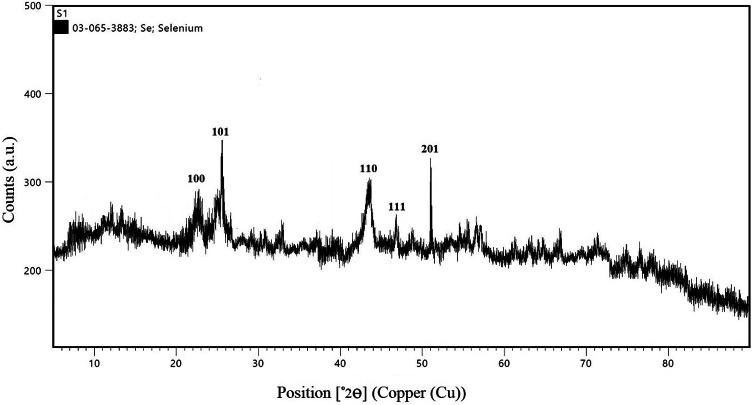
FTIR spectra of *D. pinnata L* tuberous roots Phyto-synthesized (SeNPs).

Fourier Transform Infrared (FTIR) spectroscopy identified functional groups involved in reducing and stabilizing SeNPs. Peaks at 2921 cm^–1^ corresponded to C–H stretching vibrations from aliphatic hydrocarbons, while a strong peak at 1658 cm^–1^ indicated C = O stretching, likely from carbonyl groups. Additional peaks at 1391 cm^–1^ (C–H bending or carboxylate groups) and 1125–1025 cm^–1^ (C–O stretching) confirmed the involvement of alcohols, ethers, or esters. Peaks at 525–485 cm^1^ confirmed selenium-metal bonds (Se–Se or Se–O), Fig. 5.

These findings align with Satpathy et al. (2024)^37^ who also identified similar functional groups in FTIR spectra of biogenic SeNPs. The broad FTIR peaks in this study suggest extensive biomolecular interactions with nanoparticle surfaces, emphasizing their role in stabilization Mamidi et al. (2024)^38^. Highlighted the role of phenolic compounds and flavonoids in enhancing SeNP stability and biocompatibility.

This study’s results are consistent with recent research emphasizing plant-based SeNPs synthesis. Satpathy et al. (2024)^37^, Ikram et al. (2024)^36^ and Mamidi et al. (2024)^38^. Demonstrated that variations in phytochemical composition, reaction conditions, and precursor concentrations significantly influence SeNPs size, morphology, and stability. Future research should explore optimizing these parameters to tailor SeNPs properties for applications in antimicrobial therapy, antioxidant systems, and environmental remediation.

### HPLC analysis and phenolic compound identification

**Fig. 6:**
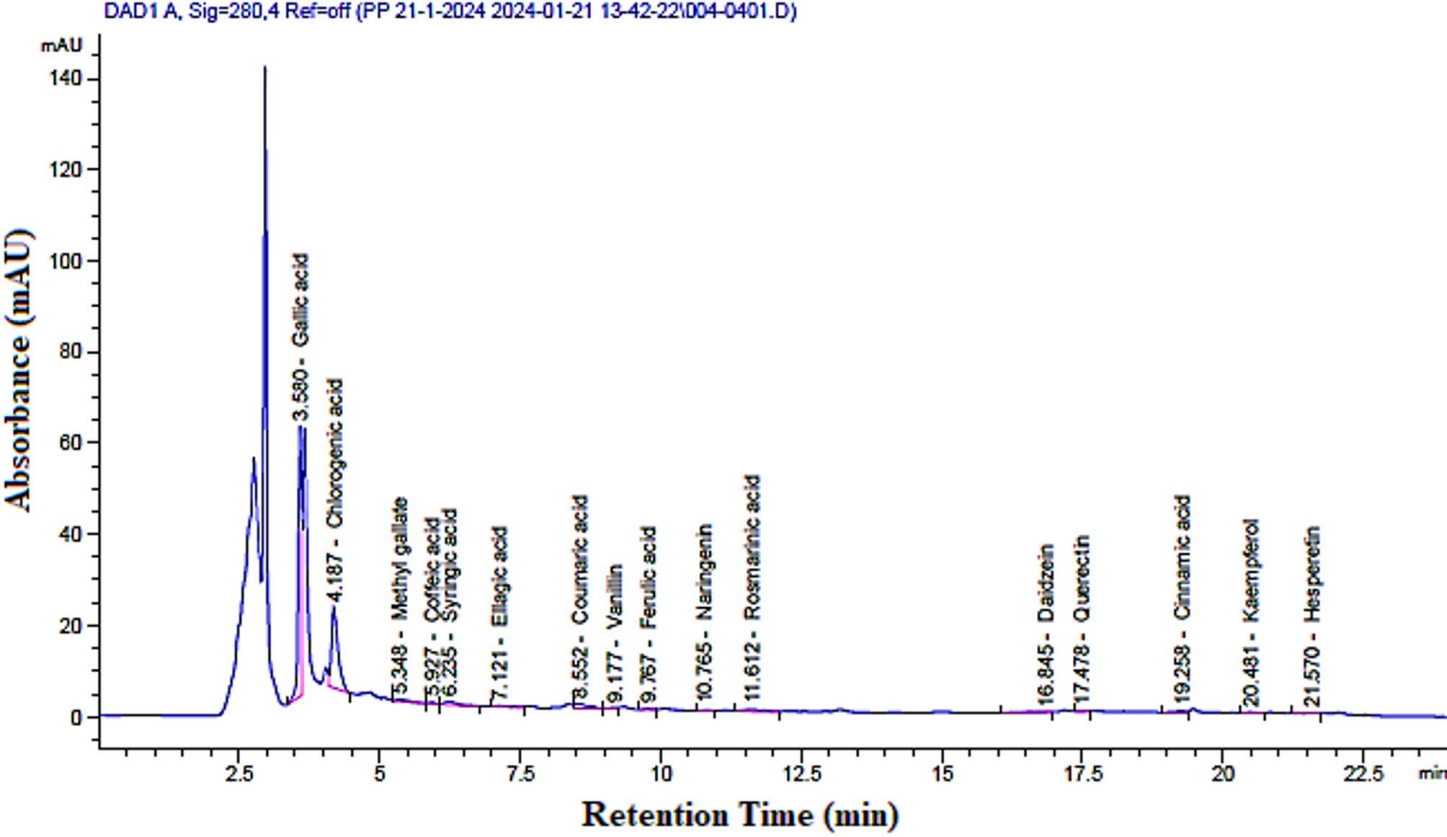
HPLC chromatogram of phenolic and flavonoid compounds in methanolic crude extract of *D. pinnata* L. tuber powder.

**Fig. 7:**
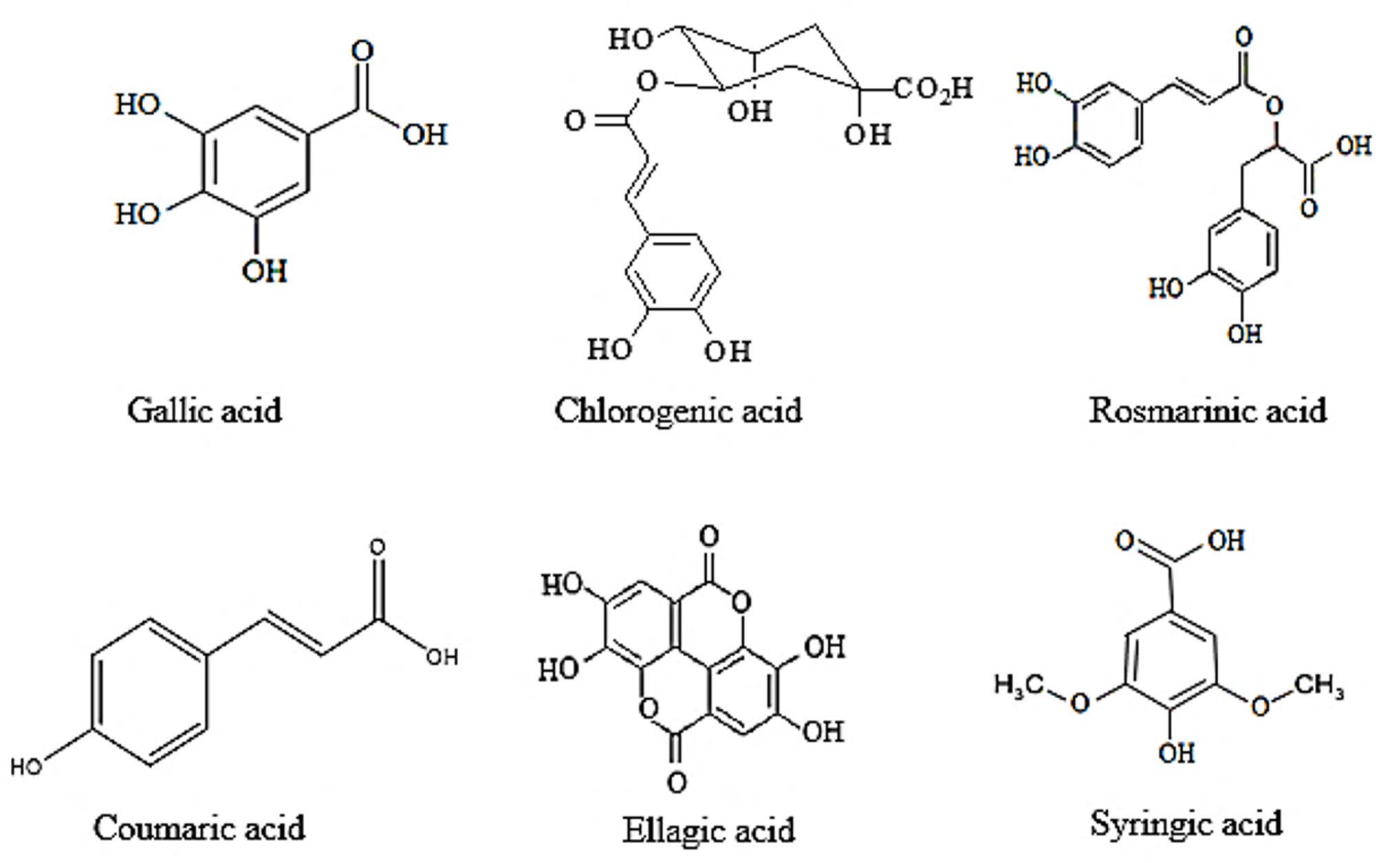
The high concentration phenolic structures of *D. pinnata L* powder was detected using HPLC.

HPLC analysis of Dahlia tuber methanol extract revealed a diverse array of phenolic and flavonoid compounds, including gallic acid, chlorogenic acid, syringic acid, caffeic acid, coumaric acid, quercetin, and kaempferol. These compounds not only acted as reducing agents in the synthesis of selenium nanoparticles (SeNPs) but also played a vital role in stabilizing them. The high concentrations of gallic acid (1070.58 µg/g) and chlorogenic acid (903.87 µg/g), both known for their strong antioxidant and reduced properties, were particularly significant in ensuring successful nanoparticle synthesis Figs. 6 and 7; Table 1. Similar findings were reported by Tritean et al. (2023)^39^who identified gallic acid as a key compound in enhancing SeNPs stability when derived from sea buckthorn leaf extract.

### Role of phenolic compounds in senps synthesis and biological activities

Phenolic and flavonoid compounds such as those found in *D. pinnata L* tuberous roots extract are widely regarded as central to biogenic nanoparticle synthesis. According to Gonzalez-Lemus et al. (2022)^40^, phenolic-rich plant extracts facilitate electron transfer to reduce metal ions, enabling the green synthesis of nanoparticles. Chlorogenic acid, for example, was highlighted by Mazhar et al. (2024)^41^ for its role in producing stable and bioactive SeNPs under optimized conditions.

The HPLC-based profiling in this study aligns with the established application of chromatography in nanoparticle research. Recent advances, as discussed by Galić et al. (2022)^42^. Emphasize the importance of phenolic compound quantification to optimize green synthesis protocols. This approach not only ensures the reproducibility of nanoparticle properties but also aids in understanding the interactions between phytochemicals and nanoparticles.

The identified phenolic and flavonoid compounds exhibit diverse biological activities that enhance the functional potential of SeNPs: Gallic Acid: A strong antioxidant with anticancer and antimicrobial properties, it stabilizes nanoparticles and enhances their therapeutic efficacy (Tritean et al., 2023)^39^. Chlorogenic Acid: Noted for its neuroprotective and antidiabetic effects, it supports nanoparticle stability and extends potential applications in metabolic disorders (Mazhar et al., 2024)^41^. Syringic Acid: Exhibits antioxidant and anti-inflammatory properties, contributing to the functional versatility of SeNPs^40^. Quercetin and Kaempferol: These flavonoids are widely recognized for their antiviral and anti-inflammatory effects, improving the biocompatibility of SeNPs (Ravi et al., 2024)^43^.

The composition of *Dahlia* tuber extract shows similarities with other plant extracts used in SeNPs synthesis but also presents unique advantages due to the high concentrations of gallic acid and chlorogenic acid. This distinct profile suggests its potential as a superior reducing and stabilizing agent compared to other plant-derived systems. Studies by Albqmi et al. (2023)^44^ and Mohammed et al. (2024) further reinforce the role of phenolic compounds in tailoring nanoparticle properties, including size, morphology, and bioactivity.

**Table 1:** Analyzing the phenolic and flavonoid components in the methanol extract of *D. pinnata L* tuberous roots powder using high-performance liquid chromatography.

Compounds	Conc. (µg/g)	Retention time (min.)
Gallic acid	1070.59	3.58
Chlorogenic acid	903.87	4.187
Methyl gallate	11.9	5.348
Caffeic acid	10.19	5.927
Syringic acid	37.14	6.235
Ellagic acid	24.35	7.121
Coumaric acid	33.35	8.552
Vanillin	5.21	9.177
Ferulic acid	10.69	9.767
Naringenin	7.68	10.765
Rosmarinic acid	53.57	11.612
Daidzein	8.7	16.845
Quercetin	6.0	17.478
Cinnamic acid	4.09	19.258
Kaempferol	7.32	20.481
Hesperetin	3.98	21.57

### Biological activities

#### Isolate collection and identification

In this study, 70 *E. coli* isolates were collected from laboratories from samples of diabetic patients suffering from urinary tract infections, wound infections, bloodstream infections, and respiratory tract infections, and were identified as described in the method section. The sources of these samples included urine (40%), wound swabs (35%), blood (15%), and sputum (10%). Based on their antimicrobial resistance profiles, the isolates were classified into two groups: multidrug-resistant (MDR) isolates and non-MDR isolates. Among them, 15 isolates (21.4%) were identified as MDR, showing resistance to critical antibiotics such as β-lactams, glycopeptides, macrolides, and tetracyclines. The remaining 55 isolates (78.6%) were classified as non-MDR, as they retained susceptibility to most first-line antibiotics, allowing for conventional treatment options.

#### Antimicrobial susceptibility profiles

**Fig. 8:**
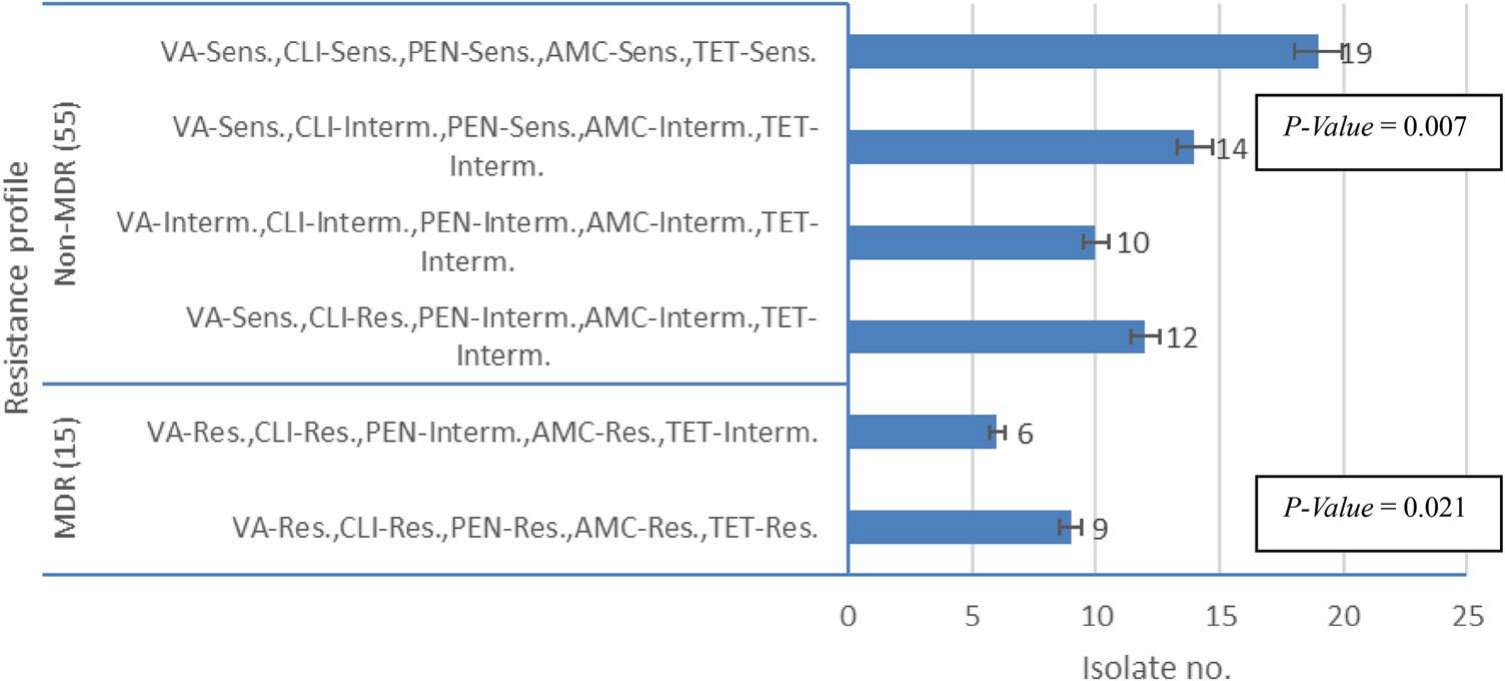
Antimicrobial Susceptibility Profiles between MDR and non-MDR *E. coli* isolates from diabetic patients.

The antimicrobial susceptibility profiles revealed distinct differences between MDR and non-MDR *E. coli* isolates. Among the 15 MDR isolates, 9 were completely resistant to vancomycin (VA), clindamycin (CLI), penicillin (PEN), amoxicillin-clavulanate (AMC), and tetracycline (TET). The remaining 6 MDR isolates exhibited intermediate resistance to AMC and TET while maintaining resistance to VA, CLI, and PEN. In contrast, the non-MDR isolates (*n* = 55) demonstrated a more varied susceptibility profile. Twelve isolates were resistant to CLI and showed intermediate susceptibility to AMC, PEN, and TET, while remaining sensitive to VA. Fourteen isolates displayed sensitivity to most antibiotics, except for intermediate susceptibility to CLI. Additionally, 19 non-MDR isolates showed full susceptibility to all tested antibiotics Fig. 8.

#### MIC and MBC results of selenium nanoparticles and selenium precursor

The antimicrobial efficacy of selenium nanoparticles (SeNPs), selenium precursors, and vancomycin was assessed against both MDR and non-MDR bacterial isolates by determining the Minimum Inhibitory Concentration (MIC) and Minimum Bactericidal Concentration (MBC). SeNPs demonstrated significantly superior activity compared to selenium precursors and vancomycin.

For MDR isolates, SeNPs exhibited MIC values ranging from 25 to 50 µg/ml and MBC values from 50 to 100 µg/ml, with mean MIC and MBC values of 35 ± 12 µg/ml and 76.6 ± 26 µg/ml, respectively. In non-MDR isolates, SeNPs showed even greater efficacy, with MIC values between 10 and 25 µg/ml and MBC values between 25 and 50 µg/ml, averaging 15 ± 4.5 µg/ml (MIC) and 35 ± 12 µg/ml (MBC). In contrast, selenium precursors required higher concentrations for comparable effects, emphasizing the enhanced bioactivity of SeNPs. Vancomycin showed limited efficacy, with MIC values of 8 to 16 µg/ml and MBC values of 16 to 32 µg/ml, particularly against MDR isolates Table 2.

**Table 2:** The antimicrobial efficacy of senps, selenium precursors, and reference antibiotics against MDR and non-MDR isolates is summarized below.

Isolate group	Agent	MIC range (µg/ml)	MBC range (µg/ml)	Mean MIC ± SD (µg/ml)	*P* Value	Mean MBC ± SD ( µg/ml)	*P* Value
MDR (*n* = 15)	SeNPs	25–50	50–100	35 ± 12	< 0.005	76.6 ± 26	< 0.005
	Selenium Precursor	150–250	200–300	190 ± 34		250 ± 56	
	Vancomycin	8–16	16–32	12 ± 3		24.5 ± 8	
Non-MDR (*n* = 55)	SeNPs	10–25	25–50	15 ± 4.5	< 0.005	35 ± 12	< 0.005
	Selenium Precursor	75–150	100–200	120 ± 28		160 ± 35	
	Vancomycin	1–8	2–16	5 ± 1.9		10 ± 44	

Interpretation: MDR isolates required significantly higher SeNPs and selenium precursor concentrations to inhibit and kill bacteria compared to non-MDR isolates. SeNPs showed better antimicrobial activity than selenium precursors, demonstrating lower MIC and MBC values across both groups. Non-MDR isolates were more responsive, with lower MICs and MBCs.

These findings align with previous reports of the broad-spectrum antimicrobial efficacy of SeNPs. Huang et al. (2024)^45^ observed MIC values of 25–50 µg/ml, supporting the superior activity of SeNPs, which can be attributed to their size, bioavailability, and interaction with bacterial membranes. Similarly, Boroumand et al. (2019)^46^ demonstrated MIC values of 10–50 µg/ml for SeNPs, highlighting their potent effects against MDR strains.

The synthesis method plays a pivotal role in determining SeNPs’ efficacy. In this study, the use of *D. pinnata L.* extract provided phenolic and flavonoid compounds that enhanced nanoparticle stability and bioactivity. These results are consistent with Sans-Serramitjana et al. (2023)^47^ who reported similar MIC values (10–25 µg/ml) for SeNPs synthesized with *Annona muricata* fruit extract.

Selenium precursors showed limited activity, likely due to reduced stability and interaction with bacterial cells compared to nanosized selenium. This observation aligns with Rangrazi et al. (2020)^48^ who emphasized the importance of size and surface properties in determining antimicrobial efficacy.

Vancomycin, a standard antibiotic, demonstrated relatively high MIC and MBC values against MDR isolates (8–16 µg/ml and 16–32 µg/ml, respectively). These values reflect the diminishing effectiveness of traditional antibiotics, as noted by Ridha et al. (2024)^49^. In their analysis of vancomycin’s efficacy against MDR *E. coli.*

SeNPs’ superior performance against MDR isolates (mean MIC of 35 µg/ml and MBC of 76.6 µg/ml) underscores their potential as alternative or adjunct therapies. This observation is supported by studies like Mamidi et al. (2024)^38^ which advocate combination therapies involving SeNPs to combat resistant infections effectively. Future investigations should optimize synthesis parameters to maximize antimicrobial activity while minimizing cytotoxic effects (Safaei et al., 2022)^50^.

#### α-Amylase inhibition

**Fig. 9:**
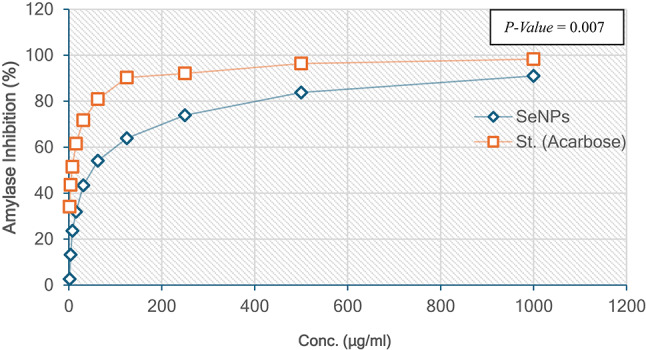
α-Amylase Inhibition percentage by Selenium Nanoparticles (SeNPs) and Acarbose.

Selenium nanoparticles (SeNPs) displayed a concentration-dependent inhibitory effect on α-amylase, with inhibition rates ranging from 2.6 to 91.0%, reaching the maximum inhibition at 1000 µg/ml, and an IC_50_ value of 50.32 µg/ml, indicating moderate efficacy​. In comparison, acarbose exhibited significantly stronger inhibition, ranging from 34.1 to 98.3%, with an IC_50_ value of 5.85 µg/ml, validating its potent antidiabetic activity (Wang et al., 2024^51^)​​. The plant extract (2CS) showed weaker inhibitory activity, with inhibition rates between 8.0% and 51.6%, and an IC_50_ value of 967.6 µg/ml, reflecting limited bioavailability of active compounds​​ Fig. 9.

#### α-Glucosidase inhibition

**Fig. 10:**
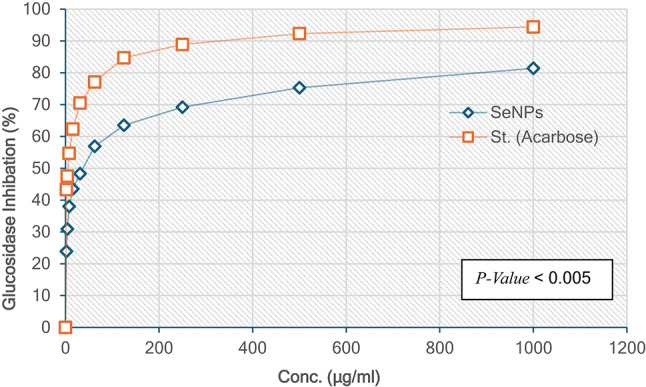
α-Glucosidase inhibition percentage by selenium nanoparticles (SeNPs) and ascorbose.

SeNPs demonstrated moderate inhibitory activity against α-glucosidase, with rates ranging from 23.9 to 81.4%, and an IC_50_ value of 31.55 µg/ml, suggesting potential utility as a supplementary antidiabetic agent​. Acarbose showed superior inhibition, with rates ranging from 43.3 to 94.4%, and an IC_50_ value of 3.93 µg/ml, reaffirming its role as an effective inhibitor in postprandial glucose management (Tang et al., 2021^52^)​. The plant extract exhibited limited activity compared to the other agents, underscoring the importance of optimization to improve its efficacy Fig. 10.

This study highlights the potential of SeNPs as a promising supplementary antidiabetic agent, demonstrating moderate inhibition against both α-amylase and α-glucosidase. Their nanoscale properties, including a high surface area and enhanced interaction with enzymes, contribute to their inhibitory effects (Khan et al., 2023^53^; Zhao et al., 2023^54^)​​. Although less effective than acarbose, which exhibited potent inhibition with IC_50_ values of 5.85 µg/ml for α-amylase and 3.93 µg/ml for α-glucosidase, SeNPs remain a valuable candidate for further development (Wang et al., 2024^50^; Tang et al., 2021^51^)​​.

The plant extract (2CS) demonstrated weak inhibition against α-amylase, with an IC_50_ value of 967.6 µg/ml, indicating limited efficacy in its crude form. This finding emphasizes the need for advanced extraction and fractionation techniques to concentrate bioactive compounds, as these processes could significantly enhance their antidiabetic potential (Dong et al., 2024^55^; Mollania & Sahabi, 2022^56^)​​.

#### Hemolysis Inhibition by selenium nanoparticles (Se-Nano)

**Fig. 11:**
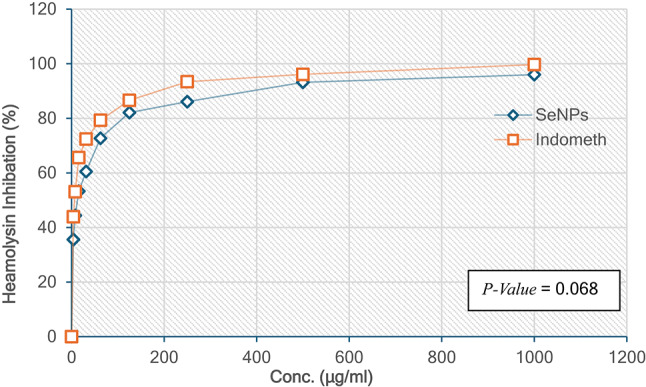
Hemolysin Inhibition percentage by Selenium Nanoparticles (SeNPs) and Indometh.

Selenium nanoparticles (Se-Nano) exhibited dose-dependent hemolysis inhibition in the hypotonic solution assay. At the highest concentration of 1000 µg/ml, Se-Nano achieved a 96.0% hemolysis inhibition, which progressively decreased at lower concentrations, reaching 35.6% inhibition at 3.9 µg/ml. The IC_50_ value was calculated as 11.53 µg/ml, indicating moderate anti-inflammatory potential through membrane stabilization. By comparison, indomethacin, the reference standard, displayed superior efficacy with 99.7% inhibition at 1000 µg/ml and 43.9% inhibition at 3.9 µg/ml, corresponding to an IC_50_ of 4.51 µg/ml. These results suggest that while Se-Nano has notable anti-inflammatory activity, its potency is less than that of indomethacin Fig. 11.

The variation in hemolysis inhibition between Se-Nano and indomethacin may be due to differences in their mechanisms of action. Indomethacin, a cyclooxygenase inhibitor, effectively stabilizes red blood cell membranes, likely due to its pharmacological optimization (Wang et al., 2024^57^). In contrast, Se-Nano’s moderate activity may result from its nanoscale size and the degree of interaction with cell membranes (Abdelghany et al., 2023^58^). The findings align with studies that emphasize the potential of selenium nanoparticles as anti-inflammatory agents through their ability to stabilize cellular membranes (Hashem et al., 2023^59^).

The synthesis method has a significant influence on the efficacy of Se-Nano. Functionalization with bioactive compounds, such as flavonoids or polyphenols, has been shown to enhance the anti-inflammatory activity of nanoparticles. For instance, Omar et al. (2023)^60^ Demonstrated that selenium nanoparticles stabilized with quercetin exhibited improved hemolysis inhibition, highlighting the role of synthesis in optimizing their biological effects. Similarly, Abdelghany et al. (2023)^58^ Reported enhanced anti-inflammatory activity for selenium-based nanocomposites doped with glycogen.

## Conclusion

This study demonstrates the successful green synthesis of selenium nanoparticles (SeNPs) using *D. pinnata* L tuber extract, emphasizing its eco-friendly and sustainable nature. The synthesized SeNPs were comprehensively characterized, confirming their nanoscale properties, unique trigonal crystal structure, and bioactive surface functionalization attributed to plant-derived compounds, including gallic and chlorogenic acids.

The antimicrobial activity of SeNPs was particularly noteworthy, exhibiting potent efficacy against *E. coli* isolates, including multidrug-resistant strains. The enhanced activity compared to selenium precursors and conventional antibiotics highlights their potential as an alternative antimicrobial agent, especially in combating resistant infections.

Moreover, the SeNPs demonstrated promising antidiabetic activity, as evidenced by their ability to inhibit key enzymes involved in glucose metabolism, α-amylase, and α-glucosidase. While not as potent as acarbose, their significant inhibitory effects underscore their therapeutic potential. Similarly, their dose-dependent anti-inflammatory activity further supports their role in mitigating inflammation, though with slightly lower potency than the standard drug indomethacin.

Overall, the bioactivities exhibited by SeNPs synthesized using *D. pinnata* L extract position them as promising candidates for diverse biomedical applications. Their natural synthesis route, combined with potent antimicrobial, antidiabetic, and anti-inflammatory properties, provides a compelling foundation for further investigation into their therapeutic potential and clinical applications. While the in vitro findings are promising, further exploration is warranted to fully understand the clinical relevance of the synthesized SeNPs. Future studies should investigate in vivo efficacy, biocompatibility, pharmacokinetics, and potential toxicity.

## Electronic supplementary material

Below is the link to the electronic supplementary material.

Supplementary Material 1

## Data Availability

The data supporting the findings of this study are available from the corresponding author upon reasonable request.
